# ID 164 - O Uso de Evidências para a Regulação Sanitária: experiência da implementação do Ciclo de Prioridades

**DOI:** 10.5327/2237-9622.2025.v34s1.164

**Published:** 2025-11-25

**Authors:** Flavia Tavares Silva Elias, Margarete Martins de Oliveira, Erica Tatiane da Silva, Erika Barbosa Camargo, Maíra Catharina Ramos

**Keywords:** Avaliação de Tecnologias em Saúde, regulação sanitária

## Abstract

**Introdução:**

A cooperação técnica-cientifica tem sido estratégia de articulação intersetorial entre ciência e tecnologia e regulação sanitária. Desde 2018 a Agência Nacional de Vigilância Sanitária (Anvisa) e a Fundação Oswaldo Cruz (Fiocruz) de Brasília atuam em avaliações de tecnologias em saúde para apoio às decisões reguladoras. Em junho de 2022, iniciou-se o Ciclo de Prioridades para estudos de síntese de evidências, de forma a identificar temas que corroborassem com a qualificação dos processos regulatórios e com o fortalecimento do papel da Anvisa na proteção e na promoção da saúde. Apesar de amplamente utilizado pelo Ministério da Saúde, o ciclo de priorização para pesquisa ainda é incipiente no campo da regulação sanitária.

**Método:**

O Ciclo de Prioridades dividiu-se em quatro etapas: i) identificação dos problemas e temas; ii) sistematização da coleta; iii) oficinas com as áreas técnicas; iv) apresentação dos resultados e encaminhamentos. Cada área técnica da Anvisa enviou temas para serem objetos de investigação de literatura e que dialogassem com a Agenda de Pesquisa da Anvisa.

**Resultados:**

Ao fim, foram identificados 29 temas diferentes, somando-se 52 estudos entre Pareceres Técnico-Científicos (n=9), informes (n=4) e sínteses de evidência (n=39). Macroagendas para dispositivos médicos e medicamentos foram as mais frequentes. Os estudos visam identificar evidências sobre vigilância pós-comercialização; definição de problemas regulatórios; seleção das melhores opções regulatórias e identificação da base legal para o problema selecionado. Até a presente data, seis sínteses de evidências, um informe técnico e cinco Pareceres Técnico-Científicos foram elaborados. Encontram-se em desenvolvimento 19 estudos, sendo os demais prospectados para início do segundo semestre deste ano. Por fim, os estudos fornecerão recomendações para orientar tomadas de decisão sobre medidas regulatórias sanitárias.

**Conclusão:**

A experiência vivenciada por meio da adoção do Ciclo de Prioridades, visando a identificar temas prioritários, deu-se com base nas reais necessidades dos tomadores de decisão, o que faz com que respostas às lacunas existentes no conhecimento possam ser apresentadas com potencial de contribuir na produção de evidências para apoiar à revisão de normas regulatórias, e monitoramento de pós-comercialização de tecnologias em saúde. Esta colaboração, para o desenvolvimento dos estudos, conta com uma rede de especialistas mobilizada para ampliar a capacidade de resposta e o conhecimento técnico-científico em apoio à tomada de decisão de regulação sanitária.

